# PD-L2 Serves as a Potential Prognostic Biomarker That Correlates With Immune Infiltration and May Predict Therapeutic Sensitivity in Lower-Grade Gliomas

**DOI:** 10.3389/fonc.2022.860640

**Published:** 2022-06-08

**Authors:** Qijun Xie, Xianlong Huang, Wu Huang, Fang Liu

**Affiliations:** Department of Neurosurgery, The affiliated Changzhou No.2 People’s Hospital of Nanjing Medical University, Changzhou, China

**Keywords:** lower-grade gliomas (LGGs), PD-L2, tumor immune microenvironment, immune infiltration, prognosis, therapeutic sensitivity

## Abstract

Although patients with lower-grade gliomas (LGGs; grades II and III) have a relatively favorable prognosis, patients frequently relapse and tend to progress to higher-grade gliomas, leading to treatment resistance, poor survival, and ultimately treatment failure. However, until now, thorough research has not yet been reported on the relationship between PD-L2 and immune infiltration and therapeutic sensitivity to immunotherapy and TMZ-based chemotherapy of LGGs. In this study, we found that the expression of PD-L2 is upregulated in glioma, with high PD-L2 expression predicting a worse prognosis. Univariate and multivariate Cox regression analysis both indicated that PD-L2 represented an independent prognostic factor with high accuracy in survival prediction for LGGs. A nomogram comprising of age, grade, IDH mutation, and PD-L2 was established for predicting OS. Additionally, PD-L2 was found to be remarkably correlated with immune infiltration and some anti-tumor immune functions. The degree of PD-L2 expression was also found to be strongly related to the prediction of therapeutic sensitivity to immunotherapy and TMZ-based chemotherapy. Furthermore, immunohistochemistry demonstrated that PD-L2 and the macrophage biomarker CD68 were both increased in glioma, with PD-L2 expression having a strong positive connection with CD68 expression. Taken together, PD-L2 is a prognostic biomarker for LGGs patients that may provide novel insights into glioma individualized therapeutic strategies and guide effective immunotherapy and chemotherapy.

## Introduction

Gliomas are the most prevalent and fatal primary malignant intracranial tumors of the central nervous system (CNS), with a poor prognosis and fast progression (1, 2). According to the World Health Organization (WHO) classification criterion, gliomas can be classified as grade I-IV based on histopathological characteristics and biological behaviors (3). Lower-grade gliomas (LGGs) are classified as WHO grades II and III, while glioblastoma multiforme is classified as WHO grade IV (GBM) (4, 5). Despite the recent multimodal therapeutic strategies that have yielded some recent advances, including surgical resection, radio-chemotherapy, and immunotherapy, the survival and prognosis of glioma patients are still dismal with a 5-year survival rate of 20-30% (6–8). Studies have shown that the median survival time of LGGs is highly variable, and its biological behavior shows great intrinsic heterogeneity, which can rapidly develop into high-grade glioblastoma (GBM) (9, 10). Novel and effective prognostic biomarkers for early diagnosis, prognosis evaluation, and treatment response prediction of LGGs patients are urgently needed due to the lack of accurate and effective approaches to predict the prognosis of LGGs patients.

PD-L2 is the second ligand for PD-1, with a two- to six-fold affinity for PD-1 compared to PD-L1, and it can be produced by stromal, immune, or tumor cells (11).PD-L2 is a less studied PD-1 ligand, which plays an important role in cancer progression and immune regulation. Recent studies have linked PD-L2 expression to a poor prognosis in a variety of malignancies, such as lung adenocarcinoma (12), esophageal cancer (13), renal cell carcinoma (14), gastric cancer (15), colorectal cancer (16). Multiple recent studies have revealed that the tumor immune microenvironment (TIME) works as an immunosuppressive therapeutic barrier and is increasingly recognized as a crucial regulator of tumorigenesis, progression, maintenance, and therapy resistance (17–20). Tumor-infiltrating immune cells (TIICs), such as tumor-associated macrophages (TAMs), may be able to predict cancer prognosis and the efficacy of chemotherapy and immunotherapy in the TIME, which might be a viable target for anti-cancer therapy (21, 22). Quantifying tumor-infiltrating immune cells and revealing the significance of tumor immune microenvironment components and signatures may thus aid in better understanding their role in tumor immune escape, predicting patient prognosis, and guiding the development of innovative therapeutic strategies (23). A growing body of research suggests that transcriptional signatures could well evaluate the tumor immune microenvironment and predict clinical prognosis in glioma (24, 25). Nevertheless, until now, the literature lacks a comprehensive analysis of the association between PD-L2 and infiltrating immune cells in the LGGs TME, as well as the relationship between PD-L2 and therapeutic sensitivity to immunotherapy and chemotherapy.

In this study, we comprehensively analyzed the expression pattern and prognostic value of PD-L2 in glioma with RNA-seq data and corresponding clinical data from The Cancer Genome Atlas (TCGA) (n = 529) and the Chinese Glioma Genome Atlas (CGGA) (n = 443) datasets. Furthermore, an accurate nomogram integrating PD-L2, age, grade, and IDH mutation status was constructed to predict the 1-, 3- and 5-year OS of patients with LGGs, which revealed a high efficacy for prognosis prediction. The relationship between PD-L2 and tumor-infiltrating immune cells (TIICs) infiltration in the tumor immune microenvironment (TIME) was then investigated. Subsequently, we further explored the potential biological processes and pathways of PD-L2 involvement in the pathogenesis of LGGs. Furthermore, the predictive value of PD-L2 in the efficacy of therapeutic sensitivity to immunotherapy and chemotherapy was also investigated. Our findings could shed light on the critical role of PD-L2 as a novel biomarker and potential therapeutic target in LGGs, as well as provide an underlying mechanism between PD-L2 and tumor-immune interactions.

## Materials and Methods

### Ethics Statement

The experiments were undertaken with the understanding and written consent of each subject. The study was approved by the Ethical Review Boards of the Affiliated Changzhou No. 2 People’s Hospital of Nanjing Medical University and conformed to the standards set by the Declaration of Helsinki.

### Gene Expression Data Acquisition and Analysis

Glioma tissues (WHO grade II and grade III, n=20) and normal brain tissue (n=2) embedded in paraffin were collected from patients undergoing surgery in the Department of Neurosurgery, The Affiliated Changzhou No. 2 People’s Hospital of Nanjing Medical University. The RNA-seq data and corresponding clinical data of LGG patients were downloaded from The Cancer Genome Atlas (TCGA; http://cancergenome.nih.gov/) and the Chinese Glioma Genome Atlas (CGGA, http://www.cgga.org.cn). The collected clinical data included age, gender, grade, IDH mutation status, 1p/19q codeletion status, and histological types.

### Evaluation of the Independent Prognostic Factor and Survival Analysis

Correlations between PD-L2 expression and the clinicopathological and molecular features were analyzed by the “ComplexHeatmap”, “ggalluvial”, and “ggpubr” R package. Univariate and multivariate Cox regression analyses were used to determine whether PD-L2 could be used as an independent prognostic factor in patients with LGGs, regardless of clinical and molecular characteristics such as age, gender, grade, IDH mutation status, 1p/19q codeletion status, and histological types. According to the median expression level of PD-L2, glioma patients were divided into high-expression and low-expression groups. The Kaplan–Meier survival analysis was performed to evaluate overall survival (OS) using the ‘survival’ R package.

### Development and Validation of the Nomogram Model

The nomogram including age, grade, IDH mutation status, and PD-L2 for prediction of 1-, 3-, and 5-years survival probability was conducted and verified in the TCGA cohort as well as CGGA cohort. The concordance index (C-index) and a calibration curve plot were then used to evaluate the nomogram’s predictive accuracy and discriminative ability.

### Infiltration Patterns in the Tumor Microenvironment

The ESTIMATE algorithm (Estimation of Stromal and Immune cells in Malignant Tumors using Expression data) was applied to calculate the immune score, stromal score, estimate score, and tumor purity based on the transcriptome profile of TCGA and CGGA LGG cohorts (26). Single-sample gene set enrichment analysis (ssGSEA) is an algorithm performed to quantify immune cell infiltration in a single sample according to the expression levels of immune cell-specific markers in the tumor microenvironment using the “limma”, “GSEABase”, and “GSVA” packages (27–29). To identify TME infiltrating immune cells, the list of immune cell-specific markers gene sets was collected from a previous study (30). The correlation between PD-L2 expression and tumor-infiltrating immune cells (TIICs) infiltration in the tumor immune microenvironment was analyzed by the ssGSEA analysis (31). To validate the accuracy of the ssGSEA, we analyzed the correlation of PD-L2 expression with the abundances of six types of immune cells (B cells, CD4^+^ T cells, CD8^+^ T cells, neutrophils, macrophages, and dendritic cells) from the online TIMER (https://cistrome.shinyapps.io/timer/) database (32). Currently acknowledged methods including TIMER, QUANTISEQ, CIBERSORT−ABS, CIBERSORT were performed to calculate the relationship between the expression of PD-L2 and the abundance of infiltrating immune cells among the LGGs samples from the TCGA dataset. The “GSVA” R package was used to compare the ssGSEA scores of immune cell infiltration and immune-related functions or pathways between high-PD-L2 and low-PD-L2 expression groups. Subsequently, the KEGG, GOBP, and hallmark pathways were used to identify the potential biological functions and pathways associated with PD-L2 expression level using the “GSVA” R package. Among them, Hallmark (h.all.v7.4.symbols.gmt), KEGG (c2.cp.kegg.v7.4.symbols.gmt), and GOBP (c5.go.bp.v7.4.symbols.gmt) gene sets were downloaded from Molecular Signatures Database (http://www.gsea-msigdb.org/). Statistical significance was determined for functional categories with an adjusted P-value < 0.05 or a false discovery rate (FDR) < 0.05.

### Therapeutic Sensitivity Prediction

Next, we further predict the therapeutic sensitivity of LGGs patients to anti-PD-1 and anti-CTLA-4 immunotherapy and chemosensitivity of temozolomide (TMZ). To better predict the therapeutic sensitivity of immune checkpoint inhibitors (ICIs), we downloaded the immune cell and immunophenotype data from The Cancer Immunome Atlas (TCIA) (https://tcia.at/home). The immunophenogram was used to predict anti-PD1/PD-L1 therapy responses in LGGs. The immunophenogram was used to calculate the immunophenoscore (IPS) among four types (CTLA4 positive + PD-1 positive, CTLA4 negative + PD-1 negative, CTLA4 positive + PD-1 negative, CTLA4 negative + PD-1 positive, CTLA4 negative + PD-1 positive) from the TCGA-GBM database. The IPS scale ranged from 0 to 10.A high PD-1 positive IPS predicts a good response to anti-PD-1/PD-L1 therapy. Furthermore, we used the R package “pRRophetic” to predict the chemosensitivity of temozolomide (TMZ) based on half-maximal inhibitory concentration (IC50) in different groups (33, 34).

### Immunohistochemistry

In formalin-fixed, paraffin-embedded surgical specimens of normal brains and different grades (WHO grade II-III) of human gliomas, immunohistochemical (IHC) staining was used to detect PD-L2 and CD68 expression. First, all paraffin specimens were cut into 4 μm thick slices, dewaxed in xylene (10 min x 2) and rehydrated with graded ethanol (100%, 95%, and 75% for 2 min, respectively), and then washed with phosphate-buffered saline (PBS; 3 times for 3 min). After antigen retrieval in 10 mM sodium citrate buffer (pH 6.0) and blocking endogenous peroxidase activity with 3% H2O2 (five minutes), the slides were incubated with 10% normal goat serum for 30 minutes at 37°C. Then the sections were incubated overnight with rabbit polyclonal anti-PD-L2 (Invitrogen, PA5-82484, 1:300), anti-CD68 (ZSGB-BIO, ZM-0464, ready-to-use) at 4°C, followed by incubation with biotinylated goat anti-rabbit immunoglobulin G (IgG) secondary antibody (ZSGB-BIO, ZDR5306, ready-to-use). The sections were then stained with DAB (3,3-diaminobenzidine), counterstained with hematoxylin, dehydrated, dried, and mounted. Three experienced pathologists were invited to evaluate all immunostained slides for IHC scores. The numbers of positively stained PD-L2 cells and CD68 cells were counted under the high-magnification lens (400×) in six randomly selected visual fields (35). The immunoreactive score (IRS) was calculated by multiplying the intensity of the staining by the percentage of positive cells. The staining intensity was scored as follows: 0 (negative), 1 (weak staining), 2 (moderate staining), and 3 (strong staining). The percentage of cells that were positive was scored as follows: 0 score (0% positive cells), 1 score (1%–25% positive cells), 2 score (26%–50% positive cells), 3 score (51%–75% positive cells), and 4 score (76%–100% positive cells) (36).

### Statistical Analysis

R (version 4.1.0) and publicly available packages were used for all statistical studies. P value<0.05 was considered statistically significant. (*P<0.05, **P<0.01, and ***P<0.001).

## Results

### PD-L2 Expression Was Upregulated in Glioma and Associated With Clinical and Molecular Characteristics

**Figure 1:**
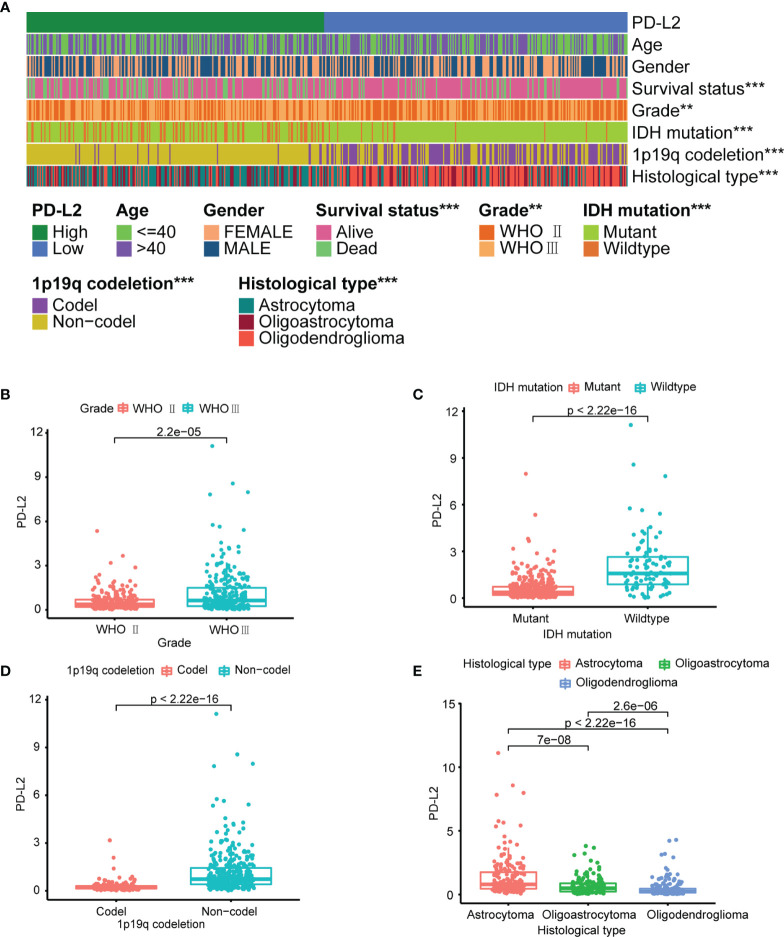
PD-L2 expression is associated with clinicopathological features in the TCGA dataset. **(A)** The heatmap showing the correlation between the expression levels of PD-L2 and the clinicopathological features including age, gender, survival status, grade, IDH mutation status, 1p/19q codeletion status, and histological types. **(B–E)** The scatter diagram showed that grade, IDH mutation status, 1p/19q codeletion status and histological types were significantly associated with PD-L2 expression (***P<0.001; **P<0.01).

The PD-L2 expression level was significantly increased with an increasing grade of glioma in the TCGA cohort **(** **Figures 1A, B** **)**. We then performed to investigate the relationship between the expression of PD-L2 and clinicopathological characteristics of LGGs patients in the TCGA and CGGA databases **(** **Table 1** **)**. The results showed that PD-L2 was significantly higher expressed in IDH wildtype than that in the IDH mutation (p< 0.001), and in 1p19q non-codeletion than 1p19q codeletion (p<0.001). Furthermore, PD-L2 expression was significantly higher in LGGs patients with a dead status than in those with an alive status(p<0.05). The expression level of PD-L2 was also found to be highly correlated with histological types (p < 0.05, **Table 1** and **Figure 1**). Consistent with the above findings, we found substantial differences between the high- and low-PD-L2 expression groups in terms of survival, grade, IDH mutation status, 1p/19q codeletion status, and histological types in the CGGA cohort **(P < 0.05,** **Supplementary Figure 1** and **Supplementary Figure 2** **)**.

**Table 1:** Correlation between PD-L2 expression and clinicopathologic characteristics of patients with LGGs in TCGA and CGGA cohorts.

Variables	TCGA				CGGA			
	Total	PD-L2^High^	PD-L2^Low^	P-Value	Total	PD-L2^High^	PD-L2^Low^	P-Value
**Age**								
<=40	248	121 (48.4%)	127 (49.8%)	0.752	189	98 (51.9%)	77 (50.7%)	0.826
>40	257	129 (51.6%)	128 (50.2%)		152	91 (48.1%)	75 (49.3)	
**Gender**								
Female	226	111 (44.4%)	115 (45.1%)	0.875	150	77 (40.7%)	73 (48.0%)	0.178
Male	279	139 (55.6%)	140 (54.9%)		191	112 (59.3%)	79 (52%)	
**Survival status**								
Alive	382	170 (68.0%)	212 (83.1%)	<0.001	173	82 (43.4%)	91 (59.9%)	0.002
Dead	123	80 (32.0%)	43 (16.9%)		168	107 (56.6%)	61 (40.1%)	
**Grade**								
WHOII	245	104 (41.6%)	141 (55.3%)	0.002	140	78 (41.3%)	62 (40.8%)	0.929
WHOIII	260	146 (58.4%)	114 (44.7%)		201	111 (58.7%)	90 (59.2%)	
**IDH mutation**								
Mutant	411	168 (67.2%)	243 (95.3%)	<0.001	261	131 (69.3%)	130 (85.5%)	<0.001
Wildtype	94	82 (32.8%)	12 (4.7%)		80	58 (30.7%)	22 (14.5%)	
**1p19q codeletion**								
Codel	167	15 (6.0%)	152 (59.6%)	<0.001	105	24 (12.7%)	81 (53.3%)	<0.001
Non-codel	338	235 (94.0%)	103 (40.4%)		236	165 (87.3%)	71 (46.7%)	
**Histological type**								
Astrocytoma	190	139 (55.6%)	51 (20.0%)	<0.001	227	152 (80.4%)	75 (49.3%)	<0.001
Oligoastrocytoma	127	65 (26.0%)	62 (24.3%)		66	19 (10.1%)	47 (30.9%)	
Oligodendroglioma	188	46 (18.4%)	142 (55.7%)		48	18 (9.5%)	30 (19.7%)	

**Figure 2:**
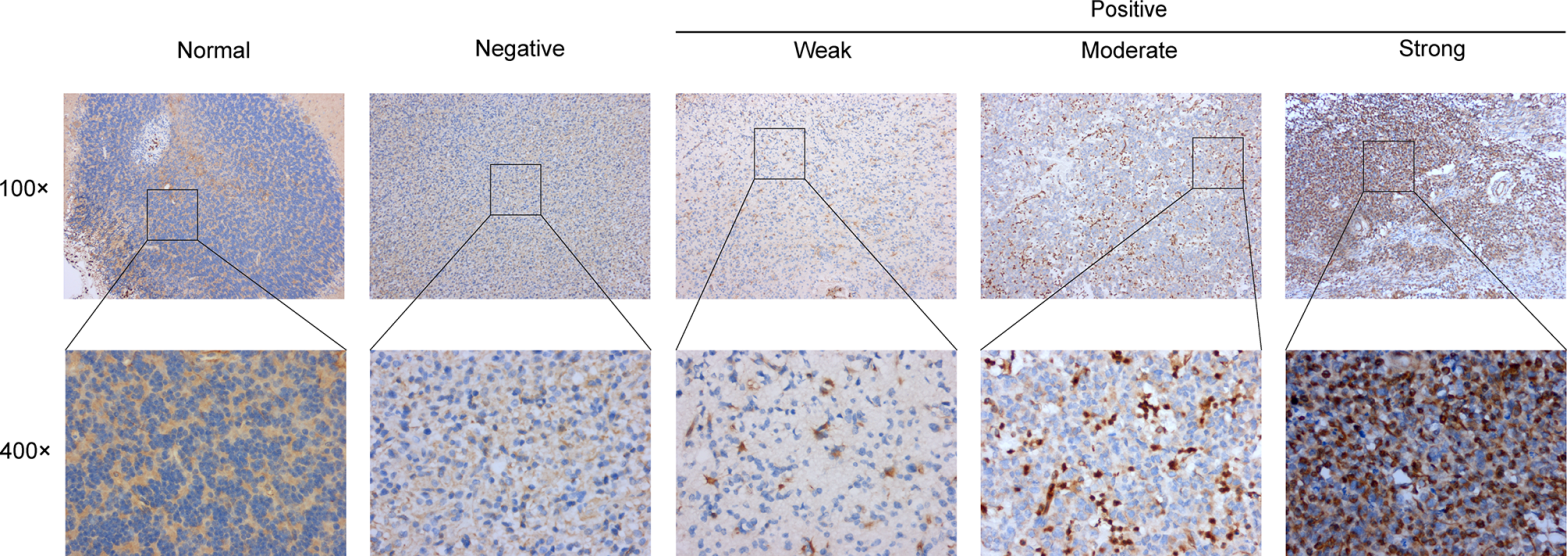
Representative images of immunohistochemical (IHC) staining of PD-L2 protein in glioma tissues and normal brain tissue (×100 and ×400).

The major clinicopathological characteristics of the 20 patients with LGGs are summarized in **Supplementary Table 1**. The average age of patients was 50 (range, 13–73 years), 15 males and 5 females. As for grade, 45% patients were in grade II. Astrocytoma was the main histological type (n = 11), followed by oligodendroglioma (n = 7) and oligoastrocytoma (n = 2). Other clinical and molecular characteristics of these patients are presented in **Supplementary Table 1**. Subsequently, the protein levels of PD-L2 were further validated *via* IHC staining in the normal brain tissue and gliomas samples from the Affiliated Changzhou No. 2 People’s Hospital of Nanjing Medical University (n=20). IHC staining of PD-L2 in tissue samples from primary gliomas WHO grade II-III patients (n = 20) and normal brain (n = 2) demonstrated that PD-L2 protein levels were more often increased in LGGs (WHO grade II-III) samples, while expression was low/undetectable in normal brain tissues **(** **Figure 2** **).** Taken together, these results indicated that PD-L2 levels were considerably higher in glioma tissue than in normal brain tissue.

### PD-L2 Was an Independent Prognostic Factor in LGGs

**Figure 3:**
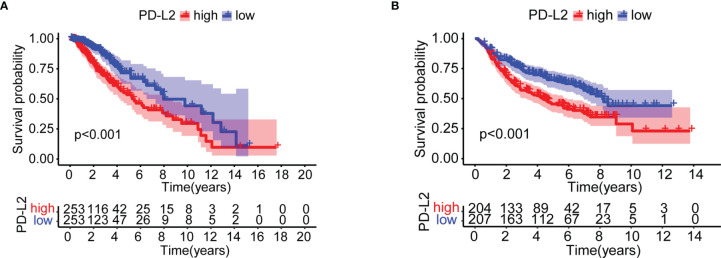
Kaplan–Meier overall survival (OS) curves for patients in the TCGA **(A)** and CGGA **(B)** datasets assigned to high- and low-expression groups.

We investigated the impact of PD-L2 expression on overall survival (OS). Patients were divided into a high expression group and a low expression group based on the median PD-L2 expression level. The Kaplan-Meier survival analysis revealed that patients with higher levels of PD-L2 expression had a worse prognosis than those with lower levels of PD-L2 expression in the TCGA and CGGA cohorts (P < 0.001, **Figures 3A, B** **)**.

**Figure 4:**
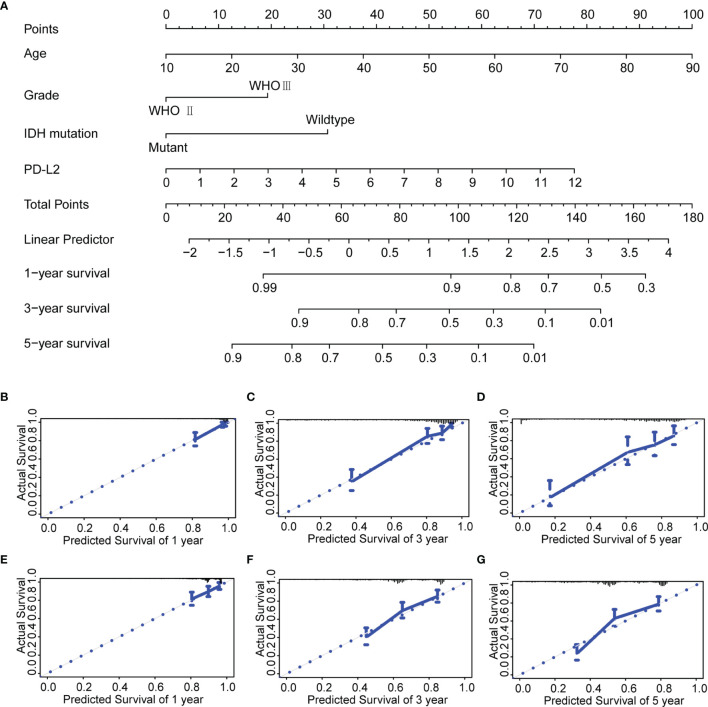
Construction and evaluation of a prognostic nomogram. **(A)** The prognostic nomogram predicts the probability of the 1-, 3-, and 5-year OS. **(B–D)** The calibration curve of the nomogram for predicting the 1-, 3-, and 5-year OS in the TCGA database. **(E–G)** The calibration curve of the nomogram for predicting the 1-, 3-, and 5-year OS in the CGGA database.

Next, to assess the independent prognostic value of PD-L2 and other clinical-pathological factors in LGGs, Univariate and multivariate Cox regression analysis indicated that PD-L2 expression remained an independent prognostic factor of OS for patients with LGGs (HR: 1.939; 95% confidence interval [CI]: 1.355-2.775) after adjusting for the six clinicopathological factors (age, gender, grade, IDH mutation status, 1p/19q codeletion status, and histological types) **(** **Table 2** **)**.We developed a nomogram model based on independent prognostic factors (age, grade, IDH mutation status, and PD-L2 expression) to quantitatively evaluate the 1-, 3-, and 5-year OS of patients with LGGs **(** **Figure 4A** **)**. The C-index of the nomogram was calculated to be 0.826, and the calibration curves for the 1-, 3- and 5-year OS had an excellent predictive effect **(** **Figures 4B–D** **).** Additionally, external validation was done by the CGGA samples. The C-index was 0.701 and the calibration curves for probabilities for 1-, 3- and 5-year OS revealed good agreement between the predicted nomogram and actual survival **(** **Figures 4E-G** **)**. Conclusively, this nomogram model based on PD-L2 expression may be a promising prognostic model for evaluating the clinical prognosis of LGGs.

**Table 2:** Univariate and multivariate analysis of association of PD-L2 and prognostic factors with overall survival in LGGs based on the TCGA and CGGA databases.

Predictor	TCGA				CGGA			
	Univariate analysis		Multivariate analysis		Univariate analysis		Multivariateanalysis	
	HR (95%CI)	P-value	HR (95% CI)	P-value	HR (95% CI)	P-value	HR (95% CI)	P-value
**Age** **(>40)**	2.889 (2.009-4.155)	<0.001	2.942 (1.890-4.580)	<0.001	1.150 (0.849-1.556)	0.367		
**Gender** **(Male)**	1.124 (0.800-1.580)	0.499			1.276 (0.936-1.739)	0.123		
**WHO grade** **(III vs II)**	3.059 (2.046-4.573)	<0.001	1.992 (1.277-3.107)	0.002	3.041 (2.128-4.347)	<0.001	3.159 (2.191-4.555)	<0.001
**IDH mutation** **(Wildtype)**	5.385 (3.777-7.679)	<0.001	2.965 (1.813-4.848)	<0.001	2.078 (1.493-2.892)	<0.001	1.573 (1.091-4.555)	0.015
**1p/19q codeletion**	0.401 (0.256-0.629)	<0.001	0.816 (0.431-1.546)	0.533	0.377 (0.256-0.555)	<0.001	0.595 (0.324-1.093)	0.094
**Histological type**	0.577 (0.392-0.848)	0.005	1.092 (0.642-1.856)	0.745	0.511 (0.394-0.662)	<0.001	0.820 (0.535-1.259)	0.365
**PD-L2**	2.794 (2.163-3.610)	<0.001	1.939 (1.355-2.775)	<0.001	1.738 (1.420-2.128)	<0.001	1.384 (1.097-1.747)	0.006

### PD-L2 Expression Correlated With Immune Cell Infiltration and Tumor Immune Microenvironment

**Figure 5:**
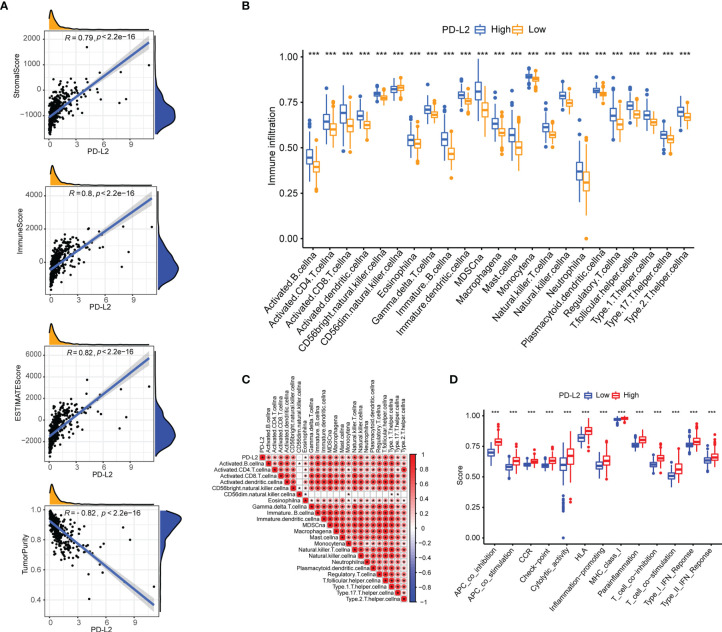
The association of PD-L2 with the tumor immune microenvironment and immune status in the TCGA cohort. **(A)** PD-L2 was positively correlated with the stroma score, immune score, and ESTIMATE score, as well as negatively correlated with tumor purity in the TCGA cohort. **(B)** The abundance of tumor-infiltrating immune cells in the high-PD-L2 and low-PD-L2 expression groups. **(C)** Pearson correlation analysis ofPD-L2 expression and immune cell infiltration. **(D)** The immune pathway functions between the high- and low- expression patients. ***P<0.001.

Then, using the ESTIMATE algorithm, we investigated the potential relationship between PD-L2 expression and immune infiltration and discovered that PD-L2 expression was positively correlated with the stroma score, immune score, and ESTIMATE score, as well as negatively correlated with tumor purity in the TCGA cohort **(** **Figure 5A** **)**. To further explore the immune infiltration difference of 23 immune cells and the immune status betweenthehigh-PD-L2 and low-PD-L2 expression groups in the TIME, we quantified the infiltrating scores of diverse immune cell subpopulations and the activity of immune-related functions or pathways with ssGSEA. The difference and correlation analyses revealed that the high-PD-L2 expression group was associated with more tumor-infiltrating immune cells, including CD8+ T cells, CD4+ T cells, B cells, macrophages, neutrophils, dendritic cells, regulatory T cells (Tregs), MDSCs, and natural killer T cells (NKT) (**Figures 5B, C** and **Supplementary Figure 3** **)**. Additionally, the analysis by the TIMER database showed that PD-L2 expression was negatively correlated with tumor purity and positively associated with the infiltrating levels of B cells, CD8^+^T cells, CD4^+^ T cells, macrophages, neutrophils, and dendritic cells **(** **Supplementary Figure 4** **)**. Furthermore, immune-related functions or pathways such as antigen-presenting cell (APC) co-inhibition, APC co-stimulation, chemokine receptor (CCR), Check-point, cytolytic activity, human leukocyte antigen (HLA), inflammation-promoting, MHC class I, para-inflammation, T-cell co-inhibition, T-cell co-stimulation, and type I and II interferon responses were associated with significantly higher ssGSEA scores in the high-PD-L2 expression group (P < 0.05, **Figure 5D**). Notably, we got similar results in the validation set, the results from the CGGA cohort also indicated that PD-L2 played a major role in immune cell infiltration in the tumor immune microenvironment of gliomas **(** **Supplementary Figures 5**, **6** **)**.

**Figure 6:**
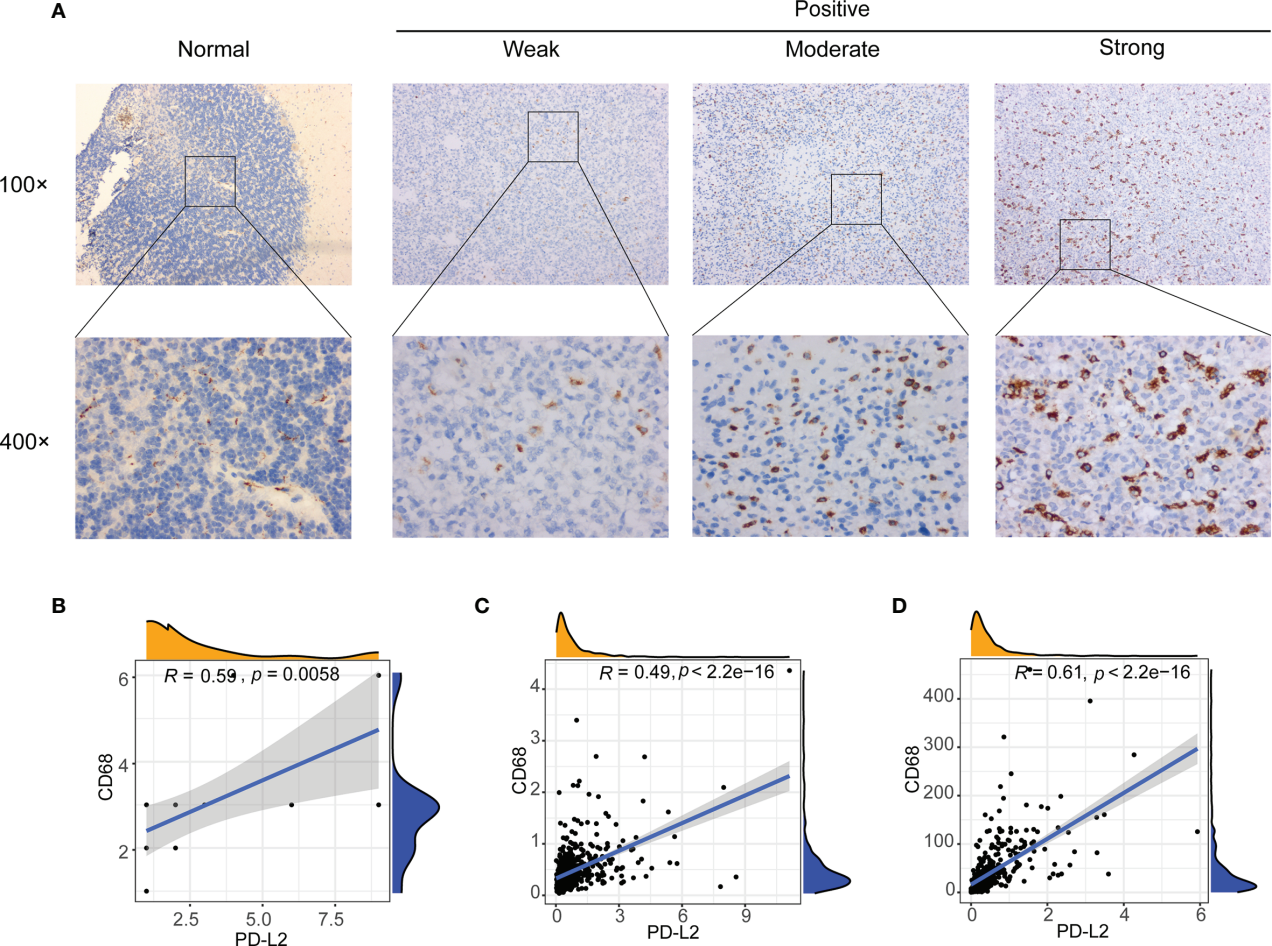
Correlation between PD-L2 expression and macrophages marker (CD68 expression) in gliomas. **(A)** Representative images of immunohistochemical (IHC) staining of CD68 protein in glioma tissues and normal brain tissue (×100 and ×400). **(B–D)** PD-L2 expression was significantly associated with macrophages marker (CD68 expression) in glioma specimens **(B)**, TCGA database **(C)**, and CGGA database **(D)**.

Our results have demonstrated that PD-L2 expression was found to be positively associated with macrophages in patients with LGGs in the TCGA (r = 0.59, P < 0.05) and CGGA cohorts (r = 0.69, P < 0.05) (**Supplementary Figures 3**, **6**) and the TIMER database’s correlation analysis showed that PD-L2 was substantially associated with macrophage (r = 0.753, P < 0.05) **(** **Supplementary Figure 4** **)**. In consistence with the findings described above, PD-L2 was more positively correlated with macrophages **(** **Supplementary Figure 7** **).** Therefore, we detected the expression of CD68 in glioma and normal brain tissues using IHC staining to further explore the relationship between PD-L2 and CD68. IHC staining results showed that the CD68 expression was observed in normal brain tissues with weak staining, while moderate or strong CD68 staining was detected in LGGs tissues (WHO II and WHO III) in almost all cases **(** **Figure 6A** **)**. Furthermore, correlation analysis revealed a positive connection between PD-L2 and CD68 (r = 0.59, P 0.05) in glioma specimens (**Figure 6B**), which was comparable with the results of correlation analysis in the TCGA and CGGA cohorts **(** **Figures 6C, D** **).** Taken together, our findings confirmed the positive correlation between PD-L2 levels and the abundance of macrophage infiltration in LGGs.

### Functional Annotation and Pathway Enrichment of PD-L2

**Figure 7:**
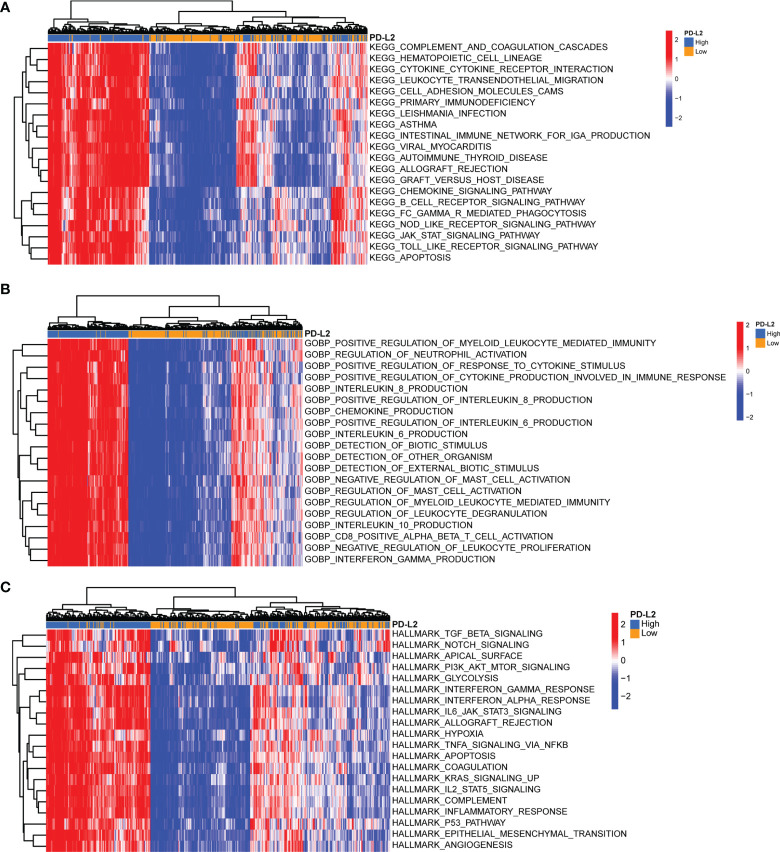
Gene Set Variation Analysis (GSVA) of biological processes and tumor-related pathways associated with the expression of PD-L2 in the TCGA cohort. Top 20 significant Kyoto Encyclopedia of Genes and Genomes (KEGG) pathways **(A)**, Gene Ontology (GO) biological processes **(B)**, and hallmark pathways **(C)** identified by GSVA analysis. GSVA, Gene Set Variation Analysis; GO, Gene Ontology; KEGG, Kyoto Encyclopedia of Genes and Genomes.

To further investigate the underlying immune-related biological processes and tumor-related pathways associated with the expression of PD-L2, we performed the KEGG, GOBP, and hallmark pathways enrichment analysis in the TCGA cohort. The findings revealed that the high-PD-L2 expression group was considerably enriched in several biological processes, including that for cytokine-cytokine receptor interaction, response to interferon−gamma, inflammatory response, angiogenesis, chemokine production, apoptosis, hypoxia, interleukin (IL)-6, and IL-8 production. Moreover, tumor-related pathways such as JAK/STAT signaling pathway, Toll-like receptor signaling pathway, PI3K/AKT/mTOR signaling, TGF-beta signaling, IL-2-STAT5 signaling pathway, IL-6-JAK-STAT3 signaling pathway, P53 pathway signaling, TNFA signaling *via* NFKB were found to be enriched in the high-PD-L2 expression group **(** **Figures 7A-C** **)**.

### Prediction of Therapeutic Sensitivity to Immunotherapy and TMZ-Based Chemotherapy

**Figure 8:**
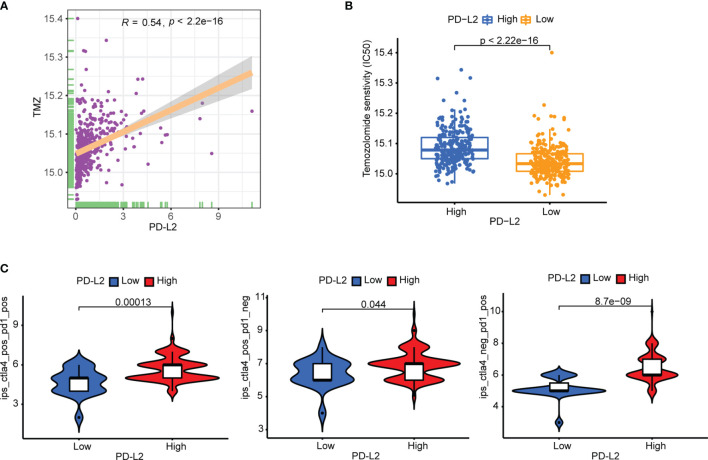
Therapeutic sensitivity prediction to immunotherapy and TMZ-based chemotherapy in the TCGA cohort. **(A)** PD-L2 was significantly positively correlated with the TMZ-based chemotherapy. **(B)** The low-PD-L2 expression patients showed a more sensitive response to TMZ-based chemotherapy based on the estimated IC50. **(C)** Patients with high PD-L2 expression had higher immunophenoscores (IPS) in three subgroups: CTLA-4 positive PD-1 positive, CTLA-4 negative PD-1 positive, and CTLA-4 positive PD-1 negative. TMZ, temozolomide; IC50, half-maximal inhibitory concentration; IPS, immunophenoscore.

Subsequently, to predict the potential chemosensitivity of temozolomide (TMZ) in LGGs, the relationship between PD-L2 expression and TMZ-based chemotherapy was explored and the results revealed a substantial positive correlation between PD-L2 expression and TMZ-based chemotherapy (r = 0.54, P < 0.05) **(** **Figure 8A** **)**. Additionally, patients in the low-expression group showed a more sensitive response to TMZ-based chemotherapy based on the estimated IC50 **(P < 0.05,** **Figure 8B** **)**. In consideration of the importance of ICIs in immunotherapy, the immunophenogram was then used to predict the response to anti-PD-1/PD-L1 therapy. We discovered that the high-PD-L2 expression group had significantly higher IPS than the low-PD-L2 expression group in the CTLA4 positive + PD-1 positive, CTLA4 positive + PD-1 negative, and CTLA4 negative + PD-1 positive types (P< 0.05) **(** **Figure 8C** **)**. These findings suggested that patients with high levels of PD-L2 expression were more likely to respond well to anti-PD-1/PD-L1 therapy or a combination of anti-PD-1/PD-L1 and anti-CTLA-4 therapy, suggesting that PD-L2 had good potential in predicting ICIs response and may benefit from immunotherapies. Conclusively, PD-L2 could contribute to the selection of optimal immunotherapy and chemotherapy strategy in glioma patients.

## Discussion

Patients with LGGs have a more favorable prognosis than those with GBM, but many advances to higher-grade gliomas, resulting in recurrence and poor survival, posing a therapeutic challenge to physicians (37–39).Despite standard treatment of maximally safe surgical resection followed by radiotherapy and adjuvant chemotherapy with temozolomide, the prognosis of glioma, particularly glioblastoma, remains poor and almost all patients relapse inevitably (40, 41). Identifying new prognostic markers of LGGs at an early stage of the tumor, thus, can successfully predict and enhance the clinical prognosis of glioma patients. A previous study showed that PD-L2 was found to be able to predict the poor prognosis of glioma patients (42, 43). However, the specific role of PD-L2 in LGGs and their therapeutic sensitivity to immunotherapy and TMZ-based chemotherapy have not been reported, and few studies have been performed to explore the relationship between PD-L2 and TIICs in the LGGs tumor immune microenvironment (TIME). In this study, we discovered that PD-L2 is elevated in LGGs samples and is substantially associated with a poor prognosis in glioma patients in the CGGA and TCGA cohorts. Univariate and multivariate Cox regression analysis confirmed that high PD-L2 expression was found to be an independent prognostic factor of patients with LGGs. Additionally, we established a nomogram that integrates PD-L2 with clinicopathological factors (age, grade, and IDH mutation status) and discovered that it performs exceptionally well in predicting 1-, 3-, and 5-year OS in glioma patients.

The role of tumor immune microenvironment in the occurrence, development, invasion, and drug-resistance of gliomas is becoming increasingly recognized (44, 45), with numerous published studies demonstrating that tumor-infiltrating immune cells (TIICs) play a critical role in the tumorigenesis, invasion, metastasis, diagnosis, treatment, and prognosis of gliomas (46–48). In our study, PD-L2 expression was found to positively correlate with the immune score, stromal score, and ESTIMATE score, as well as negatively correlate with tumor purity, indicating that high PD-L2 expression was positively correlated with immune infiltration in LGGs. Numerous studies have previously described that the role of tumor-infiltrating immune cells in various cancers is increasingly being viewed as a critical factor driving or mediating tumor progression and influencing therapeutic outcomes and patient prognosis (49, 50).Unlike a previous study (42), we further investigate the correlation between PD-L2 and TIICs *via* GSVA analysis and the online website TIMER. Notably, our results revealed that PD-L2 is closely associated with TIICs infiltration in LGGs, especially macrophages, which can be validated by the analysis of the online website TIMER.

Tumor-associated macrophages (TAMs), which play a significant role in carcinogenesis, progression, metastasis, invasion, immunosuppression, angiogenesis, and the immune response to cancer, have been linked to treatment failure and poor prognosis in LGGs patients in several recent studies. Multiple recent studies have shown that TAMs, which play an important role in tumorigenesis, progression, metastasis, invasion, immunosuppression, angiogenesis, and immune response to cancer, are linked to treatment failure and poor prognosis in LGGs patients (51, 52). Our immune cell correlation analysis revealed that PD-L2 was positively associated with macrophages in the TCGA and CGGA cohorts (r = 0.59, r = 0.69, respectively, P < 0.05). Our immunohistochemistry results are in accordance with a previous study that CD68 expression in lower-grade glioma tissues (LGGs, WHO II and WHO III) was significantly higher than the expression in normal brain tissues. Further analysis demonstrated PD-L2 expression was significantly positively correlated with macrophage infiltration in our glioma samples (r = 0.59, P < 0.05). Additionally, several lines of evidence indicate that a high TAMs infiltration, especially M2 macrophages, are closely associated with poor clinical outcomes in a wide variety of tumors and are now being recognized as potential biomarkers for diagnosis and prognosis of malignant tumors, as well as an attractive therapeutic target in cancer therapy (53–56). Chanmee et al. have reported that achieving TAMs-targeting cancer therapy by changing its polarization from M2 macrophages to M1 macrophages, inhibiting macrophage recruitment, and inhibiting the survival of TAMs, can enhance the response to treatment (57, 58).Therefore, targeting the tumor immune checkpoints and macrophages targeting may be a novel strategy for effective cancer treatments.

Immunotherapy is thought to be one of the most promising glioma treatments (59). Emerging evidence has indicated that immune checkpoint blockade (ICB) therapies such as anti-programmed death 1 (PD-1), ant-programmed cell death-ligand 1 (PD-L1) and anti-cytotoxic T lymphocyte-associated protein 4 (CTLA-4) have emerged as a promising approach as anti-cancer immunotherapy, dramatically changing treatment in solid tumors (60–62). However, the majority of patients still showed adaptive resistance with a low response rate in most cancers, especially for tumors with a low mutational burden (63–65). As a result, to improve the clinical prognosis of cancer patients, we must investigate new targets and predict the therapeutic sensitivity of immune checkpoint inhibitors (ICIs). Here, our study indicated that patients with high expression of PD-L2 are associated with a high response rate to treatment by ICIs, suggesting that PD-L2 had good potential in predicting ICIs response and high expression of PD-L2 may more likely to yield considerable clinical benefit from immunotherapy. So far, temozolomide (TMZ), a DNA alkylating agent, has become the first-line chemotherapy for the treatment of glioma, and resistance to it posed a major challenge (66–68). Therefore, it is crucial to investigate promising and reliable biomarkers for glioma therapy that can predict potential the chemosensitivity of TMZ-based chemotherapy. We discovered that patients with a low-PD-L2 expression group exhibited a more sensitive response to TMZ-based chemotherapy based on the estimated IC50, suggesting that patients with low PD-L2 were more likely to benefit from TMZ-based chemotherapy in LGGs. Combining the therapeutic sensitivity to immunotherapy and TMZ-based chemotherapy, patients with high PD-L2 may benefit from immunotherapy, whereas patients with low PD-L2 may benefit from TMZ-based chemotherapy, which implies the possible values of PD-L2 for selecting an optimal therapeutic strategy. These findings, however, will need to be further verified in the future.

Nevertheless, there are still some limitations to the present study. Firstly, we should consider the effect of tissue heterogeneity on prognostic assessments. Secondly, although we found that PD-L2 was associated with macrophage infiltration in the IHC analysis, the potential biological mechanism and pathway of PD-L2 promoting macrophage infiltration in the glioma immune microenvironment needed to be further validated using *in-vivo* or *in-vitro* experiments. Thus, we will expand the sample size and collect more complete clinical samples for further verification in our future study. Thirdly, the present findings may be the basis for further studies to validate the outcomes, such as we can further explore the development of single-cell sequencing in glioma to provide a stronger predictive value to a large extent.

## Conclusion

In conclusion, our study demonstrates that PD-L2 is overexpressed in glioma tissues and is associated with a poor prognosis. Moreover, PD-L2 overexpression correlates with immune cell infiltration, especially macrophages. The relationship between PD-L2 and the tumor immune microenvironment, as well as therapeutic sensitivity to immunotherapy and TMZ-based chemotherapy in LGGs, has never been investigated before. More importantly, PD-L2 may be a promising predictive biomarker that contributes to the selection of optimal individualized therapeutic strategy by predicting therapeutic sensitivity to immunotherapy and TMZ-based chemotherapy for future studies of patients with LGGs.

## Data Availability Statement

The original contributions presented in the study are included in the article/**Supplementary Material**. Further inquiries can be directed to the corresponding author.

## Ethics Statement

The studies involving human participants were reviewed and approved by The affiliated Changzhou No.2 People’s Hospital of Nanjing Medical University. The patients/participants provided their written informed consent to participate in this study.

## Author Contributions

**QX** contributed to the study conceptualization, methodology, software, investigation, writing - original draft. **XH** contributed to the study writing - review & editing, data curation. **WH** contributed to the study supervision, material preparation, data collection, formal analysis, visualization. **FL** writing - review & editing, supervision, conceptualization writing - review & editing, language revision, funding acquisition. All authors contributed to the article and approved the submitted version.

## Funding

Sponsored by Key Research and Development Program of Jiangsu Province (No.BE2019652), Changzhou international cooperation program (CZ20200039) and Development Program of Changzhou City (CE20205024).

## Conflict of Interest

The authors declare that the research was conducted in the absence of any commercial or financial relationships that could be construed as a potential conflict of interest.

## Publisher’s Note

All claims expressed in this article are solely those of the authors and do not necessarily represent those of their affiliated organizations, or those of the publisher, the editors and the reviewers. Any product that may be evaluated in this article, or claim that may be made by its manufacturer, is not guaranteed or endorsed by the publisher.

## Abbreviations

CGGA, Chinese Glioma Genome Atlas; GBM, Glioblastoma; GO, Gene Ontology; GSVA, Gene Set Variation Analysis; ICIs, Immune checkpoint inhibitors; IC50, half-maximal inhibitory concentration; IHC, Immunohistochemical; IPS, Immunophenoscore; KEGG, Kyoto Encyclopedia of Genes and Genomes; LGGs, lower-grade gliomas; OS, Overall survival; ssGSEA, Single Sample Gene Set Enrichment Analysis; TCGA, The Cancer Genome Atlas; TCIA, The Cancer Immunome Atlas; TIME, Tumor immune microenvironment; TMZ, Temozolomide; TIICs, Tumor-infiltrating immune cells; TAMs, tumor-associated macrophages.
