# Supplementary material for: PD-L2 Serves as a Potential Prognostic Biomarker That Correlates With Immune Infiltration and May Predict Therapeutic Sensitivity in Lower-Grade Gliomas
Source: Front Oncol. 2022 Jun 8;12:860640. doi: 10.3389/fonc.2022.860640 (PMC9213741; doi:10.3389/fonc.2022.860640)
Supplement: Supplementary file 1 [file DataSheet_1.pdf]

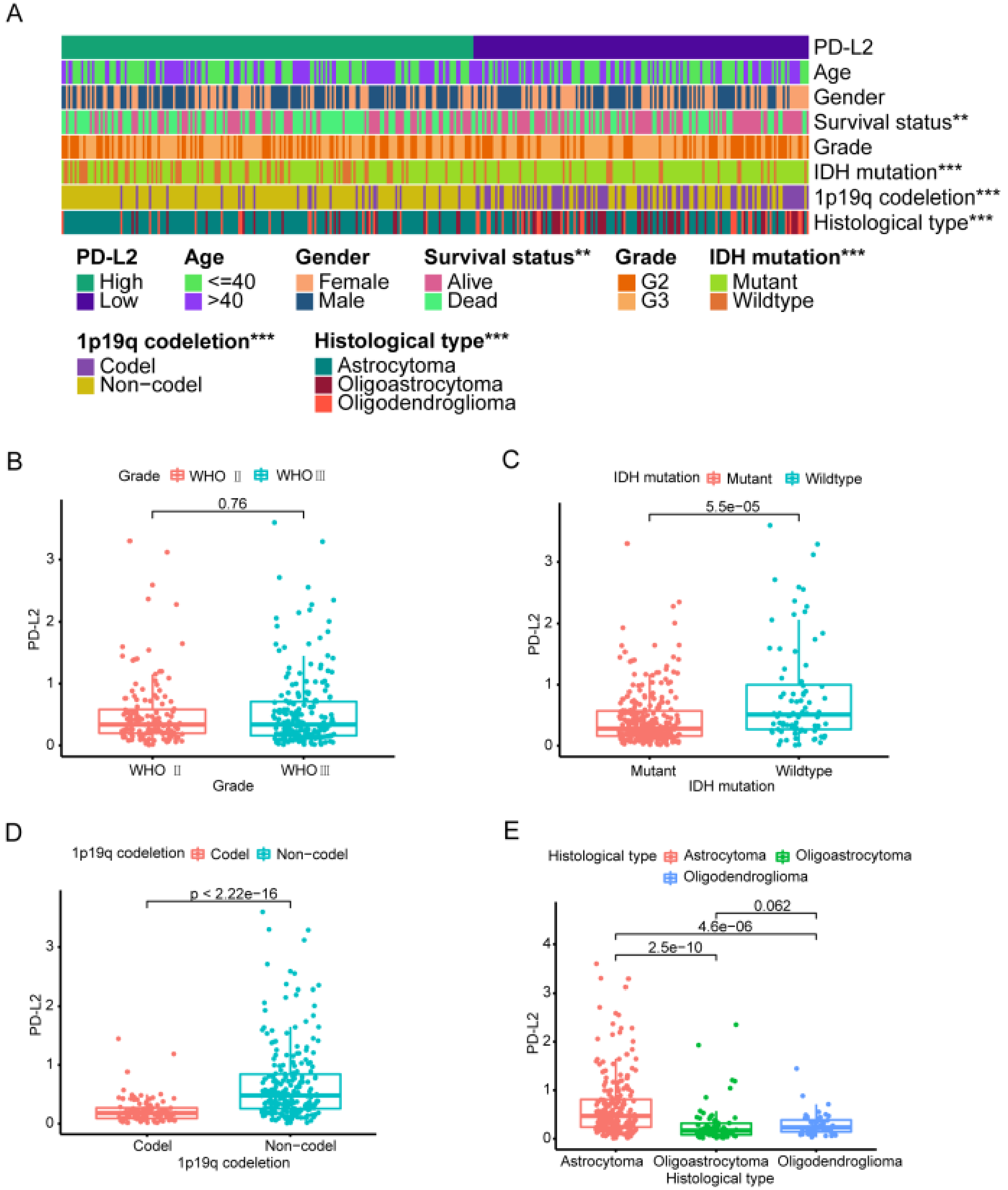

Supplementary Figure 1: PD-L2 expression is associated with clinicopathological features in the CGGA dataset. (A) The heatmap showing the correlation between the expression levels of PD-L2 and the clinicopathological features including age, gender, survival status, grade, IDH mutation status, 1p/19q codeletion status, and histological types. (B–E) The scatter diagram showed that IDH mutation status, 1p/19q codeletion status and histological types were significantly associated with PD-L2 expression (***P<0.001; **P<0.01; *P<0.05).

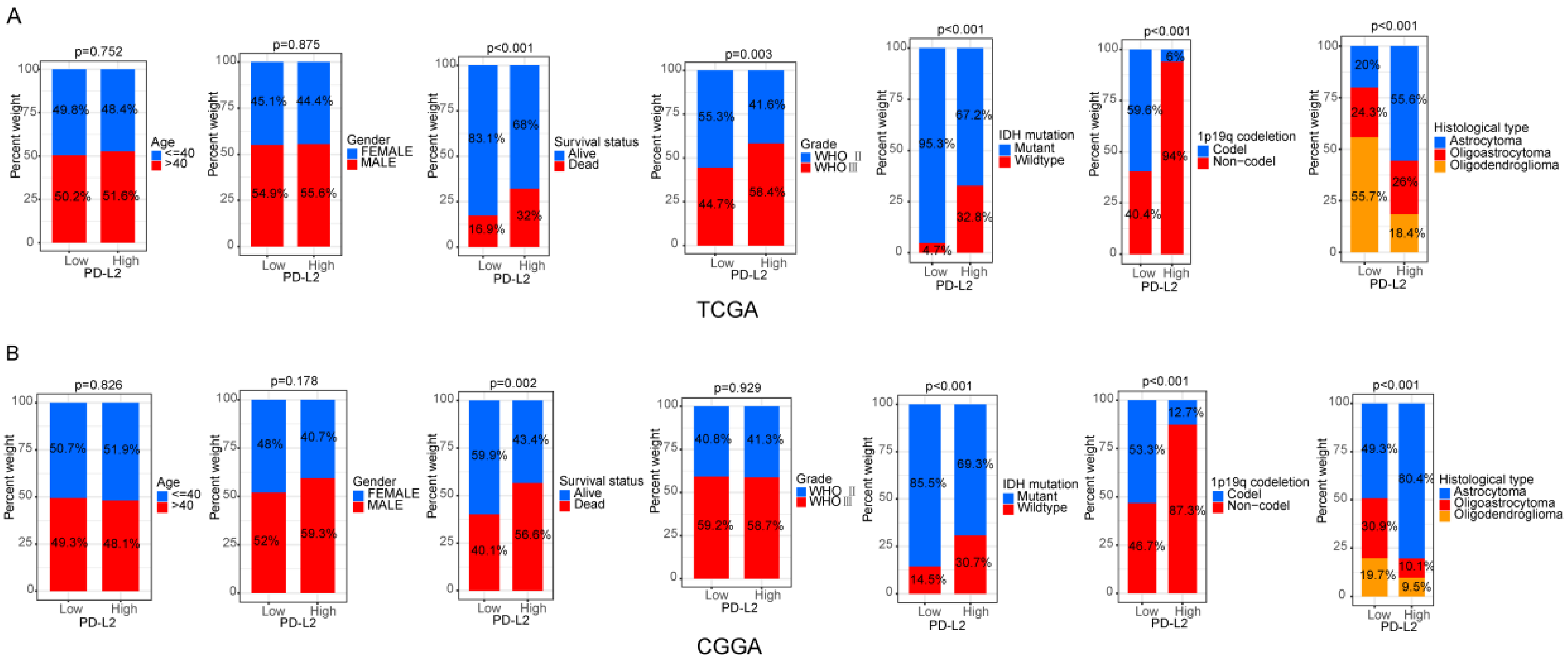

Supplementary Figure 2: The barplot representing the relative abundance (percentage) of the clinicopathological features in the high-PD-L2 and low-PD-L2 expression groups.

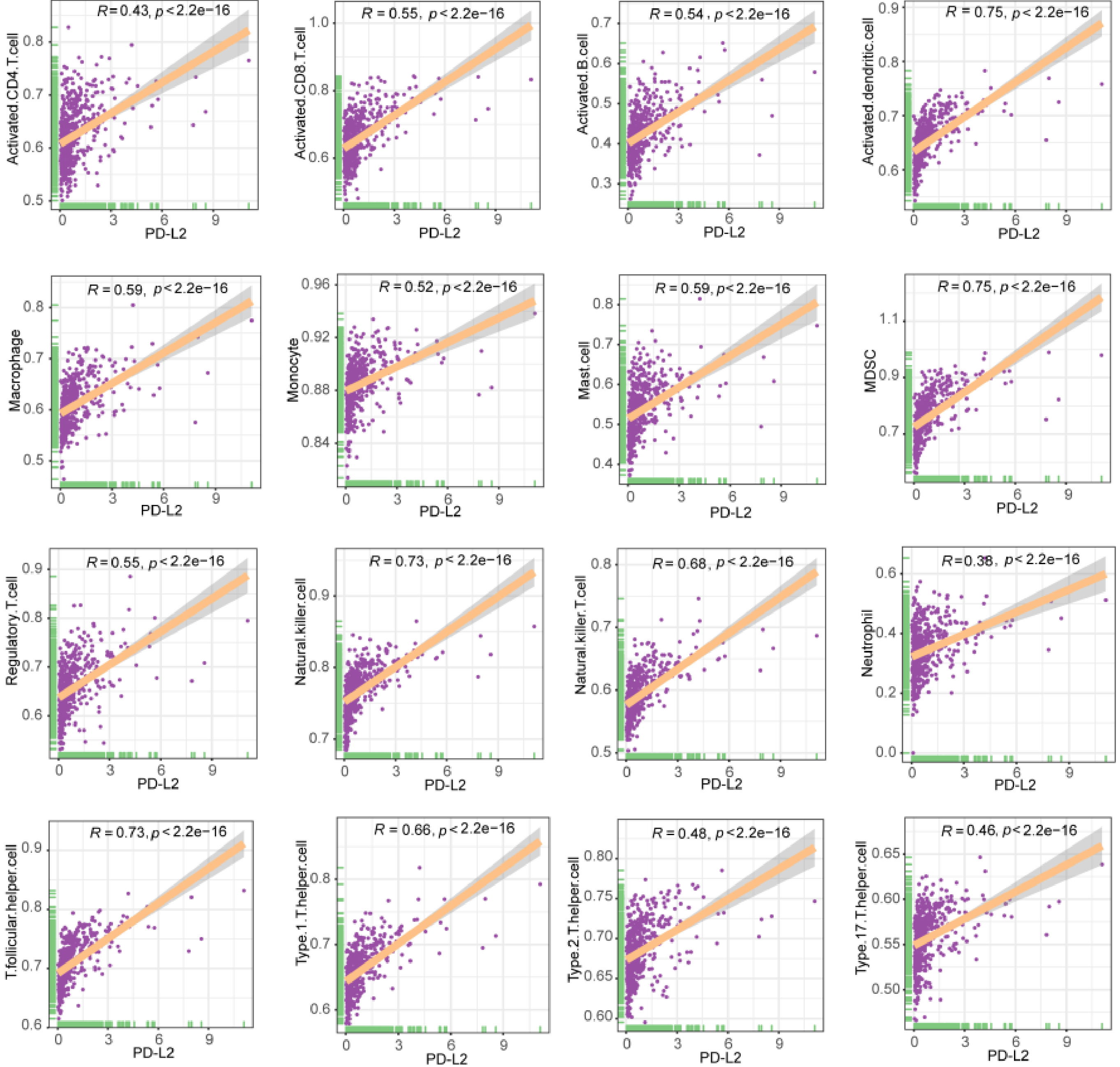

Supplementary Figure 3: Scatter plots revealed a correlation between the proportion of tumor -infiltrating immune cells and PD-L2 expression in the TCGA cohort.

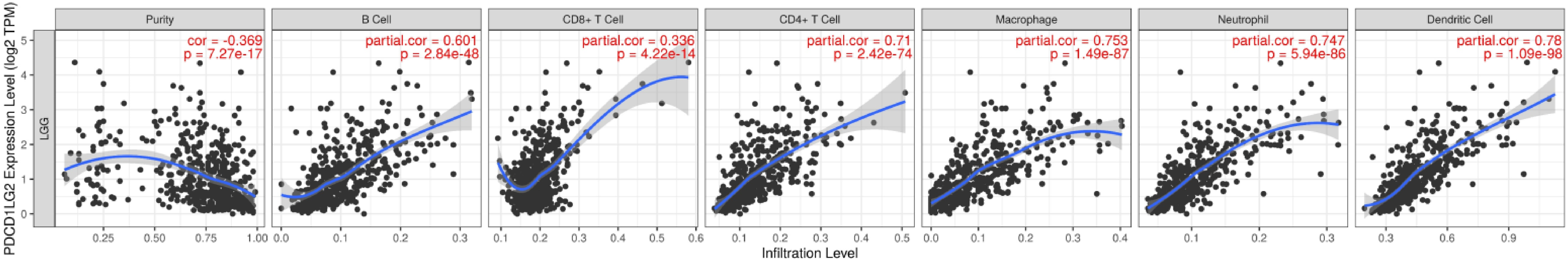

Supplementary Figure 4: The TIMER analysis was used to examine the connection between PD-L2 expression and immune infiltration levels in LGGs.

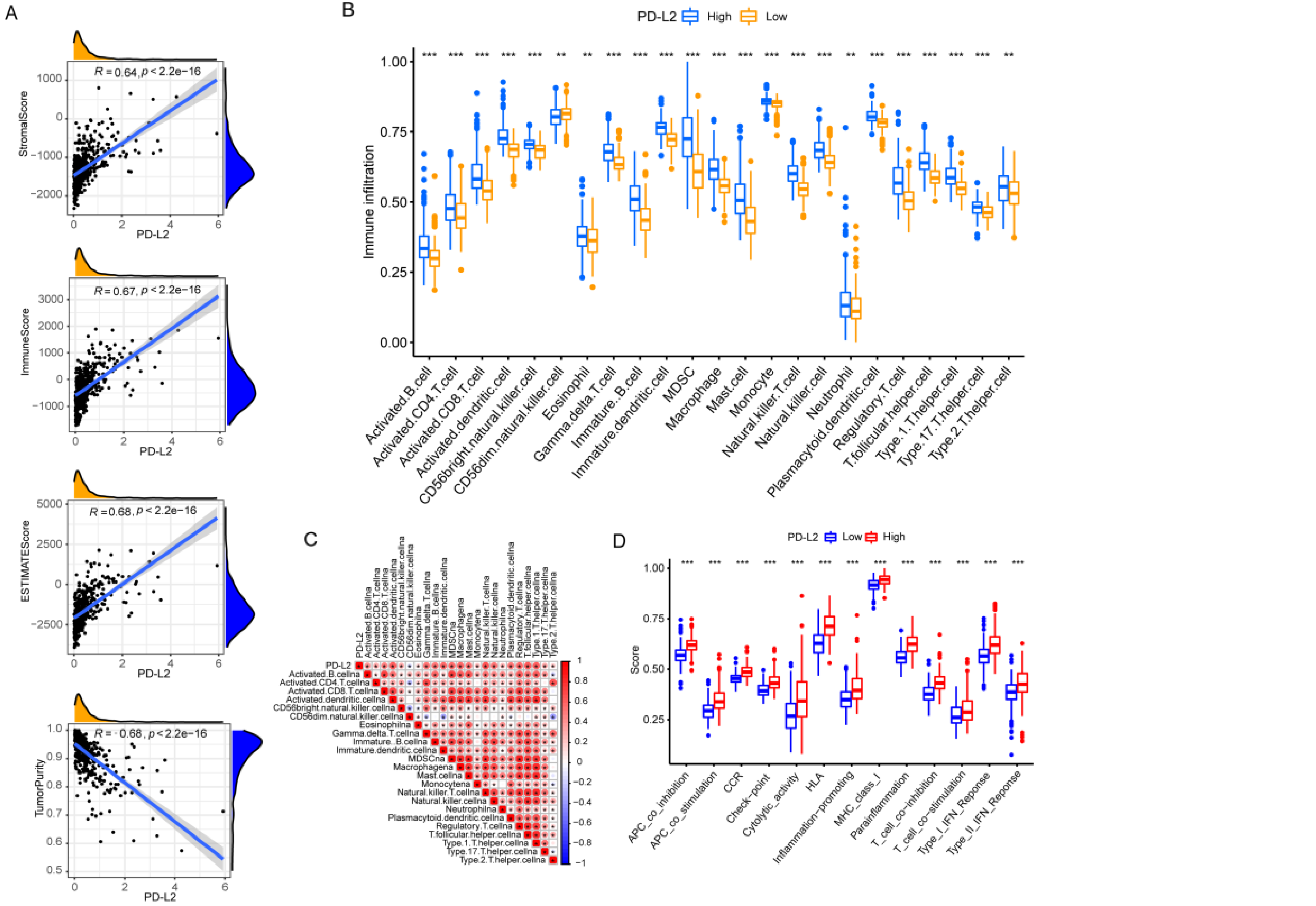

Supplementary Figure 5: The association of PD-L2 with the tumor immune microenvironment and immune status in the CGGA cohort. (A) PD-L2 was positively correlated with the stroma score, immune score, and ESTIMATE score, as well as negatively correlated with tumor purity in the CGGA cohort. (B) The abundance of tumor-infiltrating immune cells in the high-PD-L2 and low-PD-L2 expression groups. (C) Pearson correlation analysis of PD-L2 expression and immune cell infiltration. (D) The immune pathway functions between the high- and low- expression patients.

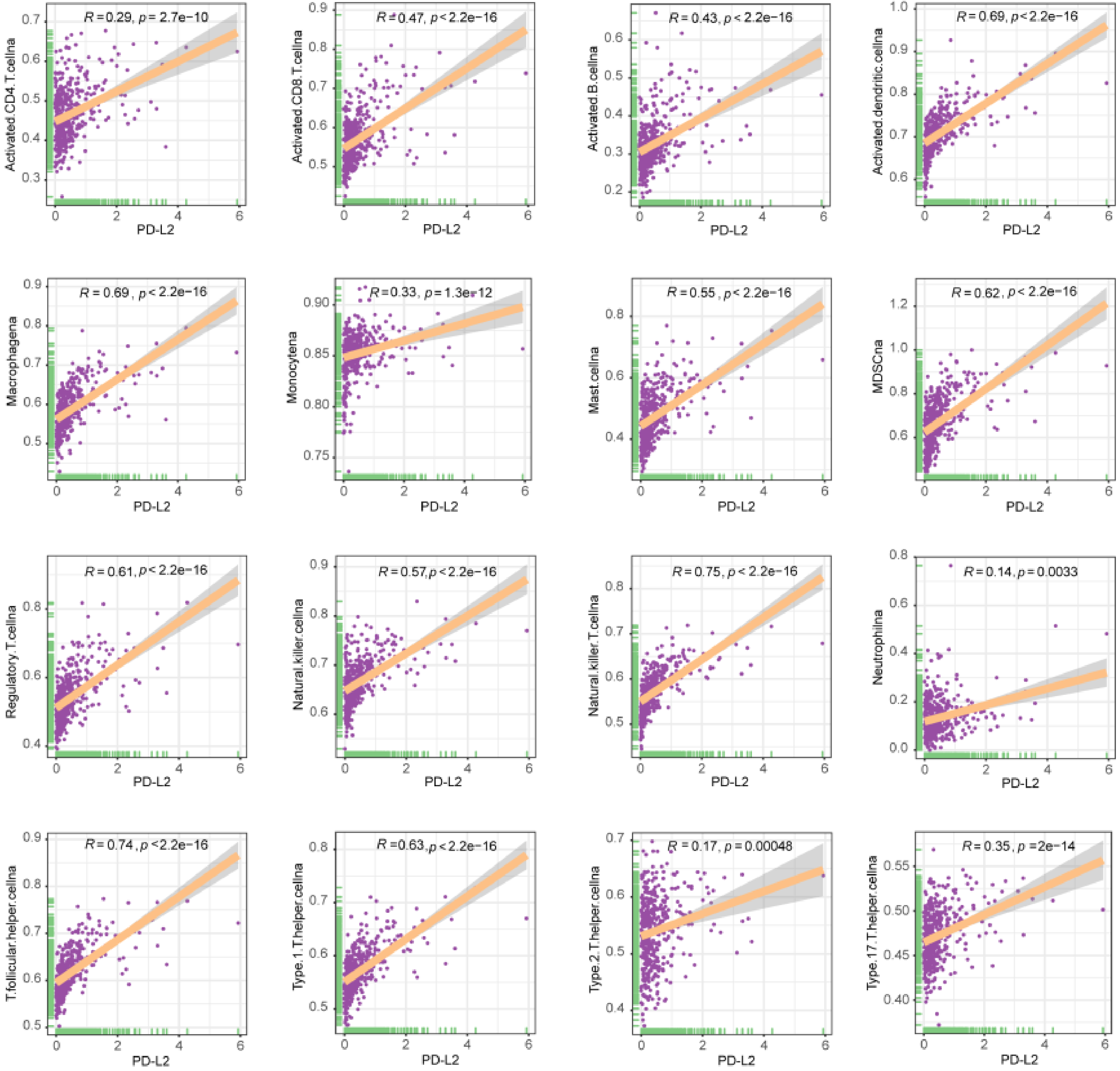

Supplementary Figure 6: Scatter plots revealed a correlation between the proportion of tumor -infiltrating immune cells and PD-L2 expression in the CGGA cohort.

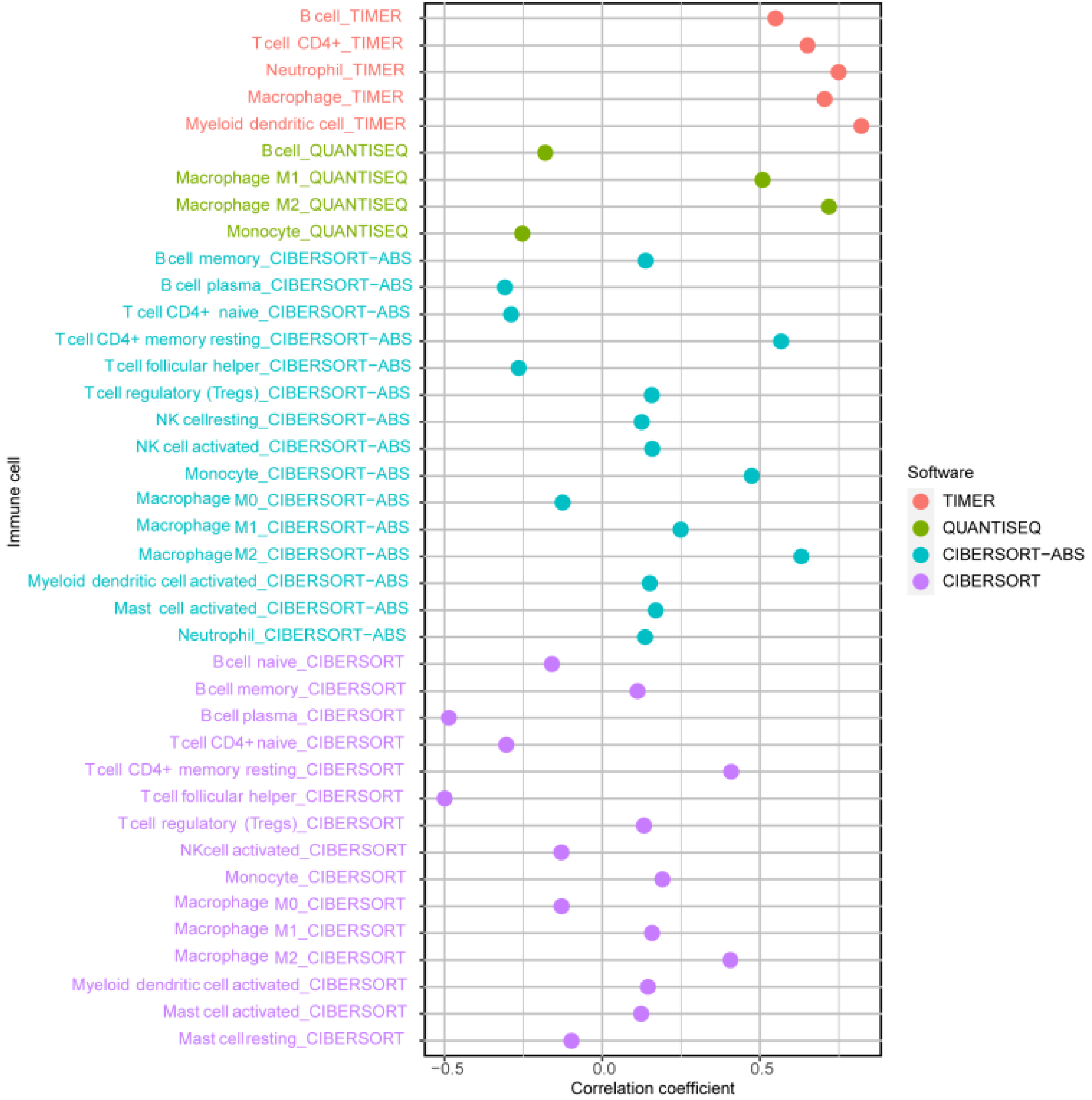

Supplementary Figure 7: Spearman correlation analysis revealed that the classifier index was positively associated with multiple tumor-infiltrating immune cells.
