# Supplementary material for: PD-L2 Serves as a Potential Prognostic Biomarker That Correlates With Immune Infiltration and May Predict Therapeutic Sensitivity in Lower-Grade Gliomas
Source: Front Oncol. 2022 Jun 8;12:860640. doi: 10.3389/fonc.2022.860640 (PMC9213741; doi:10.3389/fonc.2022.860640)
Supplement: Supplementary file 2 [file Table_1.docx]

Supplementary Table 1 Clinical and molecular characteristics of LGGs patients from Changzhou No.2 People’s Hospital.

| Patients | Age | Gender | Grade | Histological type | Lesion Location | IDH1(wildtype/mutation) | | 1p19q codeletion |
| --- | --- | --- | --- | --- | --- | --- | --- | --- |
| 1 | 51 | Male | Ⅲ | Astrocytoma | R-frontal lobe | wt | Codeletion | |
| 2 | 51 | Female | Ⅱ | Astrocytoma | L-frontal-temporal lobe | wt | Non-codeletion | |
| 3 | 28 | Male | Ⅱ | Astrocytoma | R-temporal lobe | mt | Non-codeletion | |
| 4 | 63 | Female | Ⅲ | Oligodendroglioma | L-cingulate gyrus | - | - | |
| 5 | 67 | Male | Ⅲ | Oligodendroglioma | L-frontal lobe | wt | Non-codeletion | |
| 6 | 27 | Male | Ⅱ | Oligoastrocytoma | L-frontal lobe | mt | Non-codeletion | |
| 7 | 55 | Male | Ⅲ | Oligodendroglioma | L-temporal lobe | wt | Non-codeletion | |
| 8 | 60 | Female | Ⅲ | Oligodendroglioma | L-frontal lobe | wt | Non-codeletion | |
| 9 | 62 | Male | Ⅲ | Oligodendroglioma | R-frontal lobe | - | - | |
| 10 | 55 | Male | Ⅱ | Astrocytoma | R-parieto-occipital lobe | mt | Codeletion | |
| 11 | 32 | Male | Ⅱ | Oligoastrocytoma | L-frontal lobe | mt | Codeletion | |
| 12 | 35 | Male | Ⅱ | Astrocytoma | R-temporal lobe | mt | Codeletion | |
| 13 | 71 | Female | Ⅲ | Astrocytoma | L-frontal lobe | wt | Non-codeletion | |
| 14 | 13 | Female | Ⅲ | Astrocytoma | L-thalamus-basal ganglia | wt | Non-codeletion | |
| 15 | 73 | Male | Ⅲ | Oligodendroglioma | L-frontal lobe | wt | Non-codeletion | |
| 16 | 70 | Male | Ⅱ | Astrocytoma | L-parieto lobe | - | - | |
| 17 | 40 | Male | Ⅲ | Oligodendroglioma | L-thalamus-ventricle | wt | Non-codeletion | |
| 18 | 57 | Male | Ⅱ | Astrocytoma | R-temporal lobe | - | - | |
| 19 | 57 | Male | Ⅲ | Astrocytoma | L-frontal-temporal lobe | wt | Non-codeletion | |
| 20 | 30 | Male | Ⅱ | Astrocytoma | Corpus callosum | - | - | |
